# Oral Health Status and Behavior among Cancer Survivors in Korea Using Nationwide Survey

**DOI:** 10.3390/ijerph15010014

**Published:** 2017-12-23

**Authors:** Mi Ah Han

**Affiliations:** Department of Preventive Medicine, College of Medicine, Chosun University, Gwangju 61452, Korea; mahan@chosun.ac.kr; Tel.: +82-62-230-6481

**Keywords:** health behavior, neoplasms, nutrition surveys, oral health, survivors

## Abstract

Cancer survivors remain at life-long risk of developing oral complications. This study investigated the oral health status and behavior among cancer survivors in comparison to subjects without a history of cancer using a nationwide survey. Cancer survivors and control subjects were selected from the sixth Korean National Health and Nutrition Examination Survey (2013–2015). Survivors reported chewing (34.8%) and speaking difficulties (15.3%) resulting from oral health problems. More than 36% of survivors had periodontal disease and 15.9% needed dentures. In multiple logistic regression analysis, age, household income, education level, smoking status, cancer site, and current cancer status were associated with oral health status. Approximately 43.3% of cancer survivors brushed their teeth more than three times a day. In addition, 44.9% of survivors used secondary oral products, and 30.8% had been screened for oral health problems over the past year. Age, household income and education level were associated with oral health behavior. The oral health status and behavior excepting periodontal disease and the use of secondary oral products were not significantly different between cancer survivors and controls. Although oral health status of cancer survivors was not worse than that of controls, more than half of the survivors maintained unhealthy oral practices.

## 1. Introduction

Cancer is a major global health problem and its prevalence is increasing worldwide. Based on GLOBOCAN estimates, a project of the World Health Organization, about 14.1 million new cancer cases and 8.2 million deaths occurred in 2012 worldwide [1]. With screening programs and improvement in therapy, cancer survival rate has increased dramatically since the early 1990’s in Korea [2]. Therefore, an important issue in caring for cancer survivors is how to improve their long-term quality of life. Many latent clinical disorders can emerge during or after cancer treatment that may attenuate the survival gains obtained via recent advances in curative-intent therapies. Therefore, long-term survivorship issues, including those that are related to oral health, are important components of cancer care and follow-up [3,4].

Oral health complications that are associated with cancer or cancer therapy include oral mucositis [5,6], infection [7], xerostomia [8], salivary gland dysfunction [9], taste alteration, nutritional compromise, and abnormal dental development [7]. These complications may affect oral health-related quality of life [10]. Surgical therapy of the head and neck region also could affect oral function [11] and it could lead negative psychological impacts [12].

Good oral health is important in maintaining quality of life and overall health. Chronic oral diseases have the potential to impair survivors’ quality of life after oncological treatment and are frequently neglected [4]. Further, oral health has a direct and indirect impact on systemic health, including cancer [13]. Thus, maintenance of good oral health is important for cancer patients. Cancer patients were prone to develop oral disease caused by the loss of salivary fluid secretion, difficulty of cleaning teeth due to mucositis, and limited mouth opening after surgical resection [14].

Despite worldwide recognition of the systemic health effects of cancer, oral health status and behavior among cancer survivors has not been fully investigated. To date, there are limited data on oral health status and behavior among cancer survivors. In addition, few studies have investigated oral self-care and use of dental services by cancer survivors in Korea. Therefore, the current study investigated oral health status and behavior among adults with a history of cancer utilizing data from the Korean National Health and Nutrition Examination Survey (KNHANES).

## 2. Materials and Methods 

This study utilized data from the sixth Korean National Health and Nutrition Examination Survey (KNHANES), conducted from 2013–2015 by the Korean Centers for Disease Control and Prevention (KCDC). The KNHANES study evaluated health status and related behavior at the national level using multistage probability sampling methods to select a representative cohort of non-institutionalized civilians. All of the participants in the survey participated voluntarily and written informed consent was obtained from all subjects. The Institutional Review Board of the KCDC approved survey protocol. Additional details about the study design and methods are provided in previous reports [15,16]. 

A total of 22,948 total subjects participated in this survey. Among them, 14,684 of the 18,034 adults aged 19 or older participated in both the health interview and oral health examination survey. Cancer survivors were defined as those who answered “yes” to the question, “Have you ever been told by a doctor that you had cancer?” Cancer survivors were asked about the site of their cancer, age at diagnosis, and current cancer status. The time since diagnosis was calculated as the difference between the age at the time of the survey and the age at diagnosis. The current cancer status was defined as a “yes” response to the question, “Are you currently suffering from cancer?” Control subjects were defined as subjects who reported no cancer history. Among the total 651 cancer survivors, 11 subjects with oral/head or neck cancer history were excluded. Sex and age matched controls (1:4) were selected among non-cancer subjects. Finally, 640 cancer survivors and 2560 controls were selected.

Self-rated oral health, oral health-related difficulties with chewing or speech, periodontal disease, and denture needs were investigated. Poor self-rated oral health was defined as a “poor” or “very poor” answer to the question, “How would you rate your oral health?” Chewing and speech difficulties were defined as a “difficult” or “very difficult” answer to the questions, “Do you have chewing difficulties due to oral health problems?” and “Do you have speaking difficulties due to oral health problems?”, respectively. Periodontal disease and denture needs were assessed by oral health examinations. Oral health examination was performed by well-trained public health dentists at the mobile examination center. The mouth was divided into sextants (maxillary right posterior, maxillary anterior, maxillary left posterior, mandibular right posterior, mandibular anterior, and mandibular left posterior). The periodontal disease was assessed using the community periodontal index (CPI), with 0 points (healthy), 1 point (bleeding observed, directly or using a mouth mirror, after probing), 2 points (calculus detected during probing, but all of the black band on the probe visible), 3 points (pocket 4–5 mm, gingival margin within the black band on the probe), and 4 points (pocket 6 mm or more, black band on the probe not visible). The maximal CPI score of sextants was selected and CPI score of 3 or 4 was considered to have periodontal disease. The need for dentures was assessed for maxilla and mandible (with not required, partial denture required or full denture required). If partial or full denture was needed in either maxilla or mandible, it was considered to have the denture need.

Oral health behavior was assessed by determining daily teeth brushing frequency, use of secondary oral products, and participation in oral health screenings. Teeth brushing times were recorded as before or after breakfast, lunch, dinner, after snacks, or before bedtime. The frequency of daily teeth brushing was calculated by the sum of brushings per day, and recorded as once a day or less, twice a day, or three or more times a day. Secondary oral products included dental floss, mouthwash, interdental brush, and electric toothbrush. The number of total secondary oral product use was calculated and reported as none, one, or two or more.

Demographic factors and general health behavior were used as covariates: sex, age (19–44, 45–64, ≥65 years), household income, education level (≤elementary school, middle school, ≥high school), smoking status (never, former, current), drinking frequency (none, ≤once/month, ≥twice/month), and chronic disease as self-reported by physician diagnosis (no, yes). Chronic diseases included hypertension, dyslipidemia, stroke, myocardial infarction, angina pectoris, osteoarthritis, rheumatoid arthritis, pulmonary tuberculosis, asthma, diabetes mellitus, thyroid disease, kidney failure, hepatitis B, hepatitis C, and liver cirrhosis. Household income was divided into quartiles and categorized as low, middle-low, middle-high, or high. 

General characteristics of cancer survivors and control subjects were presented as numbers and percentages. Chi-square tests were used to analyze the differences in general characteristics between groups. Oral health status and behavior between cancer survivors and controls were analyzed by chi-square tests and multiple logistic regressions. Multiple logistic regression was used to analyze the factors that were associated with oral health status and behavior among cancer survivors. SAS version 9.2 (SAS Institute, Cary, NC, USA) survey procedure was used to account for the complex sampling design with *p* < 0.05 considered to be statistically significant.

## 3. Results

Household income and education level were not significantly different between cancer survivors and controls. Current smoking and drinking rates were lower in cancer survivors compared to controls. Approximately 53% of survivors were more than 5 years from their date of cancer diagnosis and the most common cancer site was the stomach (Table 1).

Of cancer survivors, 14.8% rated their oral health as “poor”. Chewing and speech difficulties were reported in 34.8% and 15.3% of cancer survivors, respectively. In addition, 36.6% of survivors had periodontal disease and 15.9% needed dentures. Only 41.3% of survivors brushed their teeth three or more times a day. However, 44.9% of survivors used secondary oral products and 30.8% participated in oral health screenings during the past year. The prevalence of periodontal disease was significantly low in cancer survivors (adjusted odds ratio (OR) = 0.82, 95% confidence interval (CI) = 0.67–0.99) and the use of oral health examination was significantly high in cancer survivors (aOR = 1.23, 95% CI = 1.01–1.50) when compared to controls, respectively (Table 2).

Cancer survivors with lower education levels had significantly higher OR for chewing difficulties (aOR = 2.01, 95% CI = 1.22–3.31), speech difficulties (aOR = 2.87, 95% CI = 1.44–5.71), periodontal disease (aOR = 1.76, 95% CI = 1.11–2.81), and denture needs (aOR = 2.26, 95% CI = 1.23–4.17). Current smoking was associated with periodontal disease (aOR = 2.14, 95% CI = 1.01–4.52). In addition, periodontal disease and denture needs were associated with a poor oral health rating, chewing and speech difficulties due to oral health problems (Table 3).

Higher education level was associated with brushing teeth ≥3 times a day (aOR = 2.70, 95% CI = 1.67–4.37), increased use of secondary oral products (aOR = 4.60, 95% CI = 2.79–7.59), and participation in oral health screening (aOR = 3.15, 95% CI = 1.89–5.23) (Table 4).

## 4. Discussion

Although daily teeth brushing frequencies were not significantly lower in cancer survivors when compared to non-cancer controls, only less than half of them brushed their teeth more than three times a day. Daily teeth brushings more than three times a day are generally recommended to people in Korea [17]. Teeth brushing is a basic self-care behavior that is important in maintaining oral health [18] and is inversely associated with upper digestive tract cancer risk [19,20], as well as dental caries. Brushing teeth frequently, therefore, must be taken seriously for overall oral health and cancer management in daily life. 

In the present study, less than half of cancer survivors reported the use of secondary oral products. To remove plaque in interdental areas, the use of several secondary oral products is recommended. Interdental brushing, for example, is more beneficial for plaque and gingivitis prevention than teeth brushing alone [21]. In our multiple regression analyses among cancer survivors, higher levels of education and household incomes were positively associated with using secondary oral products. A lack of information and financial strain might contribute to non-use of these oral products. 

Clinically determined oral health status, such as periodontal disease and the needs of denture, was associated with self-rated oral health, chewing and speaking difficulties that are caused by oral health problems. These results suggested that clinical exam and care of oral disease might be helpful for subjective oral health.

Only 30.8% of survivors reported participating in an oral health screening within the 12 months prior to our study. In previous studies, a common reason for not seeking dental care was lack of awareness of the need for care among patients with chronic disease [22,23]. In our analysis, higher education status was associated with a higher OR for oral health screening use. Therefore, cancer survivors should be educated about the importance of regular and preventive dental care.

The prevalence of periodontal disease was significantly low, and the use of oral health screening was significantly high in cancer survivors as compared to those of controls. Periodontal disease is recognized as a possible complication of cancer and it may be exacerbated during cancer therapy [24]. Therefore, dental visits for oral and periodontal examination before and after cancer therapy are recommended [14]. They might lead to a low prevalence of periodontal disease. 

In the present study, 8.4% of survivors were current smokers and 30.8% drank alcohol more than twice per month. Although the proportion of current smokers in the cancer survivor group was lower than in the control group, current smoking was associated with periodontal disease. Cigarette smoking is a well-known risk factor for periodontal disease [25,26] and cancer prognosis [27,28]. Also, alcohol consumption is associated with periodontitis [29], and it is a risk factor for secondary cancer or recurrence in cancer patients [30,31]. Because smoking and alcohol drinking are modifiable risk factors for oral health and cancer, cancer survivors should be made aware of the health risks of these behaviors and encouraged to make healthy lifestyle choices. 

There are several limitations of the present study. First, oral health behavior measurements were collected by self-reported methods. Social desirability bias may have influenced participants to over report their health behavior. Secondly, oral health behavior could have been influenced by the attitude and knowledge of cancer survivors and the physicians who treated them, but we were not able to assess those. In a previous study, only one-third of cancer survivors indicated that they believed that cancer had affected their overall health [32]. Also, a majority of oncology nurses reported receiving less than three hours of oral health-related education or training and did not have a clinical requirement regarding oral health status assessment during their nursing school education [33]. Third, the oral function, such as salivary flow, could not be investigated due to lack of information. Another limitation of this study was that the KNHANES-selected subjects utilized non-institutionalized civilians using cross-sectional design. Therefore, cancer survivors who were admitted to hospitals and convalescent homes were not eligible to participate in KNHANES. The proportion of long-term survivors in this study may be higher than that in the general cancer survivor population, which includes survivors who have shorter survival periods. Finally, information related to cancer treatments was lacking. Current cancer status and therapy modalities can influence oral health. Also, visiting physicians for cancer treatment is able to influence oral health behavior through receiving physicians’ recommendations. Nevertheless, the strengths of this study included; this study was performed on population based nationwide survey and various oral health and behavior were investigated using interviews and oral health examinations.

## 5. Conclusions

Overall oral health status of cancer survivors was similar with that of controls, excepting the periodontal disease and the use of oral health examination. Less than half of cancer survivors brushed their teeth more than three times per day, used secondary oral products, and had oral health examinations. Cancer survivors should be educated about the association between oral health risks and cancer and the importance of preventive dental care. As some survivors did not utilize regular dental care, all health care professionals should screen their patients for general health, including oral health. In addition, health care professionals should also recommend dental care visits and adherence to good oral health behavior, which might be helpful in combatting their higher risk of oral diseases.

## Figures and Tables

**Table 1:** Characteristics of cancer survivors and control subjects.

Characteristics	Self-Reported History of Cancer (*n =* 640)	No Reported Cancer History (*n* = 2560)	*p*-Value
Sex			1.000
Men	245(38.3)	980(38.3)	
Women	395(61.7)	1580(61.7)	
Age (years)			1.000
19–44	61(9.5)	244(9.5)	
45–64	260(40.6)	1040(40.6)	
≥65	319(49.8)	1276(49.8)	
Household income			0.834
Low	191(30)	761(29.9)	
Middle-low	175(27.5)	658(25.8)	
Middle-high	137(21.5)	569(22.4)	
High	134(21)	558(21.9)	
Education			0.862
≤Elementary school	253(39.8)	1005(40.7)	
Middle school	99(15.6)	366(14.8)	
≥High school	284(44.7)	1098(44.5)	
Smoking status			<0.001
Never	402(62.8)	1628(63.6)	
Former	184(28.8)	597(23.3)	
Current	54(8.4)	334(13.1)	
Drinking frequency			0.008
None	295(46.1)	1012(39.5)	
≤once/month	148(23.1)	698(27.3)	
≥twice/month	197(30.8)	850(33.2)	
Non-cancer, chronic diseases			0.002
No	207(32.3)	992(38.8)	
Yes	433(67.7)	1568(61.3)	
Age at diagnosis (years)			
19–44	161(25.2)		
45–64	337(52.7)		
≥65	141(22.1)		
Time since diagnosis (years)			
<1	81(12.7)		
<5	219(34.3)		
≥5	339(53.1)		
Cancer site			
Stomach	134(21)		
Liver	16(2.5)		
Colorectum	73(11.4)		
Breast	84(13.2)		
Cervix	78(12.2)		
Lung	17(2.7)		
Thyroid	121(18.9)		
Other	116(18.2)		
Currently suffering from cancer			
No	363(56.7)		
Yes	277(43.3)		

Data are expressed as number (%).

**Table 2:** Oral health status and behavior among cancer survivors compared to controls without a cancer history.

Characteristics	Self-Reported History of Cancer	No Reported Cancer History	*p*-Value
Oral health status			
Subjective oral health			0.831
Poor	14.8	15.4	
Fair	37.0	37.9	
Good	48.1	46.8	
aOR (95% CI) for poor oral health ^a^	1.03(0.86–1.23)	1.00	
Chewing difficulties due to oral health problems			0.781
No	65.2	64.6	
Yes	34.8	35.4	
aOR (95% CI) for chewing difficulties ^a^	0.97(0.79–1.17)	1.00	
Speech difficulties due to oral health problems			0.660
No	84.7	84.0	
Yes	15.3	16.0	
aOR (95% CI) for speech difficulties ^a^	0.92(0.71–1.18)	1.00	
Periodontal disease			0.032
No	63.4	58.7	
Yes	36.6	41.3	
aOR (95% CI) for periodontal disease ^a^	0.82(0.67–0.99)	1.00	
Denture needs			0.588
No	84.1	84.9	
Yes	15.9	15.1	
aOR (95% CI) for denture needs ^a^	1.06(0.83–1.36)	1.00	
Oral health behavior			
Daily teeth brushing frequency			0.610
Once a day or less	16.3	15.8	
Twice a day	42.5	40.8	
Three or more times a day	41.3	43.4	
aOR (95% CI) for teeth brushing ≥3 times ^a^	0.88(0.73–1.06)	1.00	
Floss use			0.119
No	83.0	85.4	
Yes	17.0	14.6	
Mouthwash use			0.704
No	80.0	80.7	
Yes	20.0	19.3	
Interdental brush			0.792
No	84.1	83.6	
Yes	15.9	16.4	
Electric toothbrush			0.012
No	94.5	96.6	
Yes	5.5	3.4	
Use of secondary oral products			0.124
0	55.2	59.3	
1	34.1	30.0	
2 or more	10.8	10.7	
aOR (95% CI) for use ^a^	1.16(0.96–1.42)	1.00	
Oral health screening use			0.037
No	69.2	73.3	
Yes	30.8	26.7	
aOR (95% CI) for oral health screening ^a^	1.23(1.01–1.50)	1.00	

aOR, adjusted odds ratio; CI, confidence interval. Data are expressed as %. ^a^ Adjusted for household income, education, smoking status, drinking frequency and chronic disease.

**Table 3:** Associated factors with oral health status among cancer survivors.

Characteristics	Poor Self-Rated Oral Health	Chewing Difficulties Due to Oral Health Problems	Speaking Difficulties Due to Oral Health Problems	Periodontal Disease	Denture Needs
Sex (/women)					
Men	1.22(0.64–2.35)	0.94(0.47–1.91)	1.11(0.44–2.83)	1.82(0.95–3.48)	1.01(0.43–2.36)
Age (/19–44 years)					
45–64	0.80(0.42–1.53)	1.97(0.79–4.91)	3.04(0.38–24.31)	1.24(0.59–2.58)	3.16(0.69–14.41)
≥65	0.68(0.32–1.42)	2.91(1.10–7.70)	5.57(0.68–45.82)	1.53(0.69–3.39)	5.56(1.17–26.38)
Household income(/high)					
Low	2.50(1.44–4.33)	1.66(0.91–3.03)	1.03(0.48–2.23)	1.56(0.90–2.69)	0.97(0.47–2.02)
Middle-low	1.40(0.83–2.34)	0.92(0.51–1.68)	0.74(0.34–1.64)	1.22(0.71–2.07)	1.05(0.51–2.16)
Middle-high	1.43(0.84–2.44)	1.40(0.76–2.58)	0.94(0.40–2.23)	1.21(0.70–2.12)	1.21(0.56–2.62)
Education(/≥high school)					
≤elementary school	1.41(0.88–2.26)	2.01(1.22–3.31)	2.87(1.44–5.71)	1.76(1.11–2.81)	2.26(1.23–4.17)
Middle school	0.73(0.42–1.27)	1.56(0.88–2.77)	2.30(1.02–5.18)	1.78(1.05–3.02)	2.31(1.15–4.62)
Smoking status (/never)					
Former	1.54(0.85–2.78)	1.42(0.75–2.69)	1.63(0.71–3.75)	1.28(0.71–2.30)	1.35(0.62–2.96)
Current	1.90(0.87–4.16)	1.75(0.78–3.94)	1.53(0.50–4.66)	2.14(1.01–4.52)	1.31(0.51–3.38)
Drinking frequency (/none)					
≤once/month	1.37(0.86–2.18)	0.62(0.37–1.05)	0.58(0.29–1.18)	0.77(0.48–1.24)	0.68(0.36–1.31)
≥twice/month	0.88(0.56–1.39)	0.90(0.55–1.45)	0.61(0.32–1.17)	0.76(0.49–1.20)	1.03(0.59–1.81)
Chronic disease (/no)					
Yes	0.76(0.50–1.14)	1.18(0.75–1.85)	1.23(0.65–2.32)	0.85(0.57–1.28)	0.89(0.52–1.54)
Time since diagnosis (/<1 years)					
<5	1.40(0.78–2.50)	0.68(0.36–1.28)	1.09(0.45–2.68)	0.87(0.48–1.56)	0.62(0.31–1.27)
≥5	1.83(0.97–3.47)	1.26(0.64–2.48)	2.07(0.82–5.22)	0.97(0.51–1.82)	0.64(0.30–1.38)
Cancer site (/stomach)					
Liver	1.52(0.48–4.79)	1.12(0.33–3.87)	1.70(0.38–7.53)	1.19(0.39–3.61)	2.15(0.50–9.22)
Colorectum	1.28(0.67–2.46)	1.16(0.59–2.28)	0.63(0.23–1.71)	0.95(0.51–1.77)	2.81(1.28–6.15)
Breast	0.98(0.49–1.95)	0.82(0.37–1.81)	1.80(0.68–4.78)	1.56(0.79–3.06)	1.24(0.44–3.52)
Cervix	1.21(0.60–2.43)	1.82(0.87–3.82)	1.21(0.45–3.21)	0.62(0.30–1.29)	2.13(0.86–5.32)
Lung	0.43(0.12–1.49)	1.47(0.44–4.86)	1.09(0.25–4.82)	0.54(0.16–1.79)	1.30(0.32–5.29)
Thyroid	1.65(0.86–3.14)	2.20(1.09–4.45)	1.51(0.59–3.85)	1.05(0.55–2.00)	2.73(1.17–6.41)
Other	0.97(0.55–1.71)	1.33(0.72–2.44)	1.72(0.79–3.73)	0.87(0.50–1.52)	2.01(0.95–4.25)
Current suffering from cancer (/no)					
Yes	2.11(1.34–3.34)	1.39(0.85–2.27)	1.67(0.89–3.14)	0.80(0.51–1.26)	0.79(0.45–1.41)
Periodontal disease (/no)					
Yes	1.93(1.34–2.80)	1.51(1.02–2.23)	0.78(0.46–1.31)	-	-
Denture needs (/no)					
Yes	2.86(1.73–4.74)	2.62(1.62–4.23)	1.91(1.07–3.42)	-	-

Data are expressed as adjusted odds ratios (95% confidence interval).

**Table 4:** Associated factors with oral health behavior among cancer survivors.

Characteristics	Toothbrushing ≥3 Times a Day	Use of Secondary Oral Products	Oral Health Screening Use
Sex (/women)			
Men	1.01(0.53–1.94)	0.51(0.26–1.02)	1.95(0.96–3.97)
Age (/≥65 years)			
19–44	2.29(1.11–4.72)	1.18(0.55–2.51)	1.41(0.68–2.91)
45–64	1.55(1.01–2.38)	1.48(0.96–2.29)	1.19(0.76–1.87)
Household income(/low)			
Middle-low	0.88(0.54–1.42)	1.35(0.82–2.22)	1.35(0.79–2.30)
Middle-high	0.69(0.40–1.17)	1.66(0.95–2.88)	1.47(0.82–2.61)
High	0.68(0.39–1.18)	2.05(1.16–3.61)	1.50(0.84–2.68)
Education (/≤elementary school)			
Middle school	1.05(0.61–1.84)	1.14(0.65–2.01)	1.82(1.01–3.28)
≥High school	2.70(1.67–4.37)	4.60(2.79–7.59)	3.15(1.89–5.23)
Smoking status (/current)			
Never	1.65(0.76–3.59)	0.47(0.21–1.04)	1.77(0.75–4.17)
Former	0.83(0.41–1.70)	0.54(0.26–1.09)	1.53(0.72–3.27)
Drinking frequency (/≥twice/month)			
None	0.99(0.63–1.55)	1.44(0.90–2.31)	1.22(0.76–1.97)
≤once/month	1.17(0.72–1.91)	1.83(1.09–3.07)	0.89(0.53–1.50)
Chronic disease (/yes)			
No	0.75(0.50–1.13)	0.69(0.45–1.06)	0.79(0.52–1.20)
Time since diagnosis (/≥5 years)			
<1	1.41(0.74–2.69)	1.32(0.68–2.55)	0.71(0.35–1.41)
<5	1.12(0.71–1.77)	0.97(0.60–1.56)	1.33(0.83–2.12)
Cancer site (/stomach)			
Liver	1.72(0.55–5.37)	0.53(0.16–1.81)	1.23(0.39–3.89)
Colorectum	1.06(0.56–2.03)	1.39(0.70–2.77)	1.00(0.50–2.03)
Breast	0.79(0.40–1.56)	1.63(0.79–3.33)	1.15(0.54–2.42)
Cervix	0.78(0.39–1.56)	1.84(0.88–3.82)	1.05(0.47–2.35)
Lung	0.41(0.08–2.01)	1.45(0.39–5.37)	1.56(0.43–5.69)
Thyroid	1.17(0.62–2.22)	1.64(0.84–3.20)	1.72(0.87–3.41)
Other	1.01(0.57–1.78)	1.02(0.56–1.86)	0.96(0.52–1.76)
Current suffering from cancer(/yes)			
No	1.44(0.91–2.27)	0.81(0.51–1.30)	1.07(0.67–1.71)
Periodontal disease (/no)			
Yes	0.77(0.53–1.11)	1.07(0.72–1.59)	0.91(0.61–1.36)
Denture needs (/no)			
Yes	1.43(0.89–2.32)	0.98(0.59–1.61)	1.01(0.59–1.74)

Data are expressed as adjusted odds ratios (95% confidence interval).
